# Construction Notes

**Published:** 1896-08-29

**Authors:** 


### CONSTRUCTION NOTES.

### Hospital Additions, Newcastle.

"The Lady Armstrong Memorial Out-Patient Department" of the Hospital for Sick Children at Newcastle was recently opened. The building, which adjoins the Manors station, is faced with red bricks, having stone dressings. The patients' entrance is by the western door, where they enter a capacious waiting-room, a waiting-room also being provided in the lower storey. The consulting-rooms adjoin the larger waiting-rooms, and the patients pass on to the dispensary, also near at hand. There is an isolation-room provided to accommodate temporarily any patients suffering from an infectious disease. The committee-room and secretary's office are located on the upper floor, where also are rooms for the caretaker. The building is warmed throughout by hot water. The building work has been carried out by Messrs. J. and W. Lowry from designs of Mr. Frank W. Rich, architect, of Newcastle.

### New Hospital at Aldershot.

The foundation stone of the above hospital was laid this month. To be built on an open, breezy site, the elevations of this hospital will be of a simple, cottage-like character. The materials which will be used in carrying out the architect's designs are red brick for the walling, with stone dressing for the porch, with red tile roofs. Accommodation is to be provided here for ten beds in three wards—a men's ward containing five beds, a women's ward containing four, and a special ward. The general plan of the hospital will be divided into two blocks, connected by a hall. The administrative block will contain the matron's and operation rooms, and the special ward with the staff bed-rooms over. Immediately behind this block and parallel with it will be the wards block, with the men and women's wards on the left and right, and the nurses' or duty-room between them, having a complete control of both. The hall connecting the blocks will be arranged for the use of convalescents, a cheerful south aspect and verandah being provided. Advantage has been taken of the sloping site to place the kitchen and offices on a lower ground floor, a lift connecting them with the upper floors. Efficient lavatory accommodation to the various departments of the building, with the sanitation on the latest of modern principles, is to form a distinct characteristic. In addition to the open fireplaces in each room, hall, or ward, arrangements have been made for a system of heating by hot water, and the construction throughout is to be of the usual type of high-class buildings of this character. The ventilation of the wards, besides windows, which are arranged with hopper heads, is to be provided for by means of ventilating radiators with silk flap inlets, the extraction of the vitiated air being by flues in the walls and through the fleches on the roofs, the motive power being obtained by Bunsen burners. The architect of the building is Mr. John Davison, A.R.I.B.A., whose plan won the award in the competition which was arranged by the committee.

